# A Renewable and Ultrasensitive Electrochemiluminescence Immunosenor Based on Magnetic RuL@SiO_2_-Au∼RuL-Ab2 Sandwich-Type Nano-Immunocomplexes

**DOI:** 10.3390/s110807749

**Published:** 2011-08-05

**Authors:** Ning Gan, Jianguo Hou, Futao Hu, Yuting Cao, Tianhua Li, Zhiyong Guo, Jun Wang

**Affiliations:** The State key Laboratory of Novel Functional Materials and Preparation Science, Faculty of Material Science and Chemical Engineering, Ningbo University, Ningbo 315211, China; E-Mails: aaaguolong@gmail.com (J.H.); hufutao@nbu.edu.cn (F.H.); caoyuting001@126.com (Y.C.); litianhua@nbu.edu.cn (T.L.); guozhiyong@nbu.edu.cn (Z.G.)

**Keywords:** [Ru(bpy)_3_]^2+^@SiO_2_-Au, alpha-fetoprotein, sandwich-type immunoreaction, screen printed carbon electrode, electrochemiluminescence

## Abstract

An ultrasensitive and renewable electrochemiluminescence (ECL) immunosensor was developed for the detection of tumor markers by combining a newly designed trace tag and streptavidin-coated magnetic particles (SCMPs). The trace tag (RuL@SiO_2_-Au∼RuL-Ab2) was prepared by loading Ru(bpy)_3_^2+^(RuL)-conjuged secondary antibodies (RuL-Ab2) on RuL@SiO_2_ (RuL-doped SiO_2_) doped Au (RuL@SiO_2_-Au). To fabricate the immunosensor, SCMPs were mixed with biotinylated AFP primary antibody (Biotin-Ab1), AFP, and RuL@SiO2-Au∼RuL-Ab2 complexes, then the resulting SCMP/Biotin-Ab1/AFP/RuL@SiO2-Au∼RuL-Ab2 (SBAR) sandwich-type immunocomplexes were absorbed on screen printed carbon electrode (SPCE) for detection. The immunocomplexes can be easily washed away from the surface of the SPCE when the magnetic field was removed, which made the immunosensor reusable. The present immunosensor showed a wide linear range of 0.05–100 ng mL^−1^ for detecting AFP, with a low detection limit of 0.02 ng mL^−1^ (defined as S/N = 3). The method takes advantage of three properties of the immunosensor: firstly, the RuL@SiO_2_-Au∼RuL-Ab2 composite exhibited dual amplification since SiO_2_ could load large amount of reporter molecules (RuL) for signal amplification. Gold particles could provide a large active surface to load more reporter molecules (RuL-Ab2). Accordingly, through the ECL response of RuL and tripropylamine (TPA), a strong ECL signal was obtained and an amplification analysis of protein interaction was achieved. Secondly, the sensor is renewable because the sandwich-type immunocomplexes can be readily absorbed or removed on the SPCE’s surface in a magnetic field. Thirdly, the SCMP modified probes can perform the rapid separation and purification of signal antibodies in a magnetic field. Thus, the present immunosensor can simultaneously realize separation, enrichment and determination. It showed potential application for the detection of AFP in human sera.

## 1. Introduction

The clinical analysis of cancer biomarkers is critical to the early diagnosis of cancer and proteomics research, which will also promote the understanding of cancer diseases’ related biological processes [1–3]. Alpha-fetoprotein (AFP) is a well-known tumor marker related to hepatocellular carcinoma (HCC) [4]. The average concentration of AFP in serum often increases under cancer conditions from 5–20 ng mL^−1^ [5]. Thus the development of ultrasensitive detecting methods for trace levels of AFP in human serum is important for the early diagnosis of cancers. Compared with conventional immunoassays such as enzyme-linked immunosorbent assay (ELISA) and chemiluminescence immunoassay, the ECL assay not only shows high sensitivity and wide dynamic concentration response range, but also is potential and spatially controlled [6–10]. Among various ECL systems, Ru(bpy)_3_^2+^-based ECL has gained importance owing to its superior properties of high sensitivity and good stability in aqueous solution under moderate conditions [11,12]. Recently, some stable and sensitive ECL biosensors based on Ru(bpy)_3_^2+^ doped SiO_2_ nanoparticles have been intensively investigated [13–15]. On one hand, SiO_2_ nanoparticles are considered a good matrix because their surfaces are easily functionalized and conjugated to bioactive molecules, which shows great potential in bioanalysis. On the other hand, the SiO_2_ matrices can resist dye molecules coming off and this increases their photostability [13,16]. In addition, gold nanoparticles (Au NPs) have gained attention in the last years due to the unique structural, electronic, optical, and catalytic properties which have made them become very attractive materials for biosensor systems and bioassays [17]. Yuan and co-workers have proposed a novel sensitive ECL immunoassay with AFP antibody labeled RuL@SiO_2_-Au as probes [18]. However it required complex modification procedures on the electrode. Furthermore the sensor cannot be renewed after the immune products are formed on electrode.

Conventional electrodes, such as glassy carbon electrode (GCE), Au electrode or magnetic carbon paste electrode (MCPE), are relatively expensive and bulky. Moreover, they are not convenient to add or remove the magnetic iron on the long cylindrical electrode to attract the magnetic nanoprobes. However, amperometric biosensors fabricated with screen printed carbon electrodes (SPCEs) have the advantages of integration of electrodes, simple manipulations, low cost and low consumption of sample which can be used for one step determination, and then discarded [19,20]. Recently, magnetic nanoparticles have also gained increasing interest and have been widely applied in immunoassays [21,22] due to their biocompatibility, superparamagnetism and good electron conductivity [23], which can simplify the process of protein immobilization and separation [24]. The magnetic nanoprobes strategy developed recently has proven to be a highly sensitive technique for detecting human tumor cells, and is especially well suited to separate and at the same time detect low-concentrations of proteins [25,26]. More importantly, the magnetic probes can be modified and removed from the surface by a magnetic field added on the flat bottom of SPCEs. All these steps can make the electrode’s surface renewable and simplify the electrode modification steps. Furthermore, if a RuL labeled antibody is employed instead of pure antibody to be labeled on RuL@SiO_2_-Au nanoparticles as probes (RuL@SiO_2_-Au∼RuL-Ab2), the relevant ECL signal could greatly improve due to the increased number of reporter molecules (RuL) that are modified on the electrode surface.

Our present work is motivated by the promising applications of the SBAR sandwich-type immunocomplexes for ultrasensitive detection of AFP and renewable surface of SPCEs. In this study, the streptavidin-coated magnetic particles (SCMP), as supporting material, not only perform the rapid separation and purification of signal antibody on magnetic field, but they also enhance the fixed capacity of Biotin-Ab1 to improve the detection range. Furthermore, the SBAR sandwich-type immunocomplexes were readily immobilized on the working electrode of SPCEs by magnets, therefore, unlike traditional electrochemical immune sensors, the electrodes do not require complex modification and cleaning. In addition, the RuL@SiO_2_-Au∼RuL-Ab2 composite exhibited dual amplification since SiO_2_ could adsorb large amount of reporter molecules (RuL) and gold particles could provide large active surface to load more reporter molecules (RuL-Ab2). Thus, the SCMP/Biotin-Ab1/AFP/RuL@SiO_2_-Au∼RuL-Ab2 (SBAR) sandwich-type immunocomplexes were easy to control and have strong ECL signals.

## 2. Experimental Section

### 2.1. Reagents and Materials

Alpha-fetoprotein antibody (Anti-AFP, 12 mg L^−1^) was from Biocell Company (Zhengzhou, China). Elecsys AFP Kits were from Roche Diagnostics GmbH. Tris(2,2’-bipyridyl)ruthenium(II) chloride Hex hydrate-(Ru(bpy)_3_Cl_2_ 6H_2_O) was from Strem Chemicals. Tripropylamine (TPA) was from Tokyo Kasei Kogyo Co, Ltd. Gold chloride (HAuCl_4_), BSA (96∼99%) and Triton X-100 (TX-100) were bought from Sinopharm Chemical Reagent Co.Ltd (Shanghai, China). Tetraethylorthosilicate (TEOS) and 3-aminopropyltriethoxysilane (APTES) were from Aladdin Chemistry Co. Ltd. Gold nanoparticles (Au-NPs, ∼16 nm in diameter) were prepared according to the procedures reported by Enüstün *et al.* [27]. Phosphate buffered solution (PBS, pH 7.4) was prepared using 0.1 M Na_2_HPO_4_, 0.1 M KH_2_PO_4_ and 0.1 M KCl. Blocking buffer solution consisted of a PBS with 3% (w/v) BSA and 0.05% (v/v) Tween 20. Washing buffer solution consisted of a PBS with 0.1 M NaCl and 0.05% (v/v) Tween 20 (PBST). All other chemicals were of analytical grade and all solutions were prepared with doubly distilled water.

### 2.2. Apparatus

ECL experiments were carried out using a MPI-B model electrochemiluminescence analyzer (Xi’an Remax Electronic Science & Technology Co. Ltd., Xi’an, China) with the voltage of the photomultiplier tube set at 800 V and initial potential = 0.0 V, high potential = 1.2 V, scan rate = 100 mV/s. A three-electrode system was used, which consists of a screen printed carbon working electrode (SPCE), a carbon auxiliary electrode and an Ag/AgCl reference electrode (DropSens Corporation, Spain). A H600 transmission electron microscope (Hitachi, Japan) was employed to characterize the nanoparticles.

### 2.3. Preparation of RuL@SiO_2_-Au Nanoparticles

**Scheme 1.**
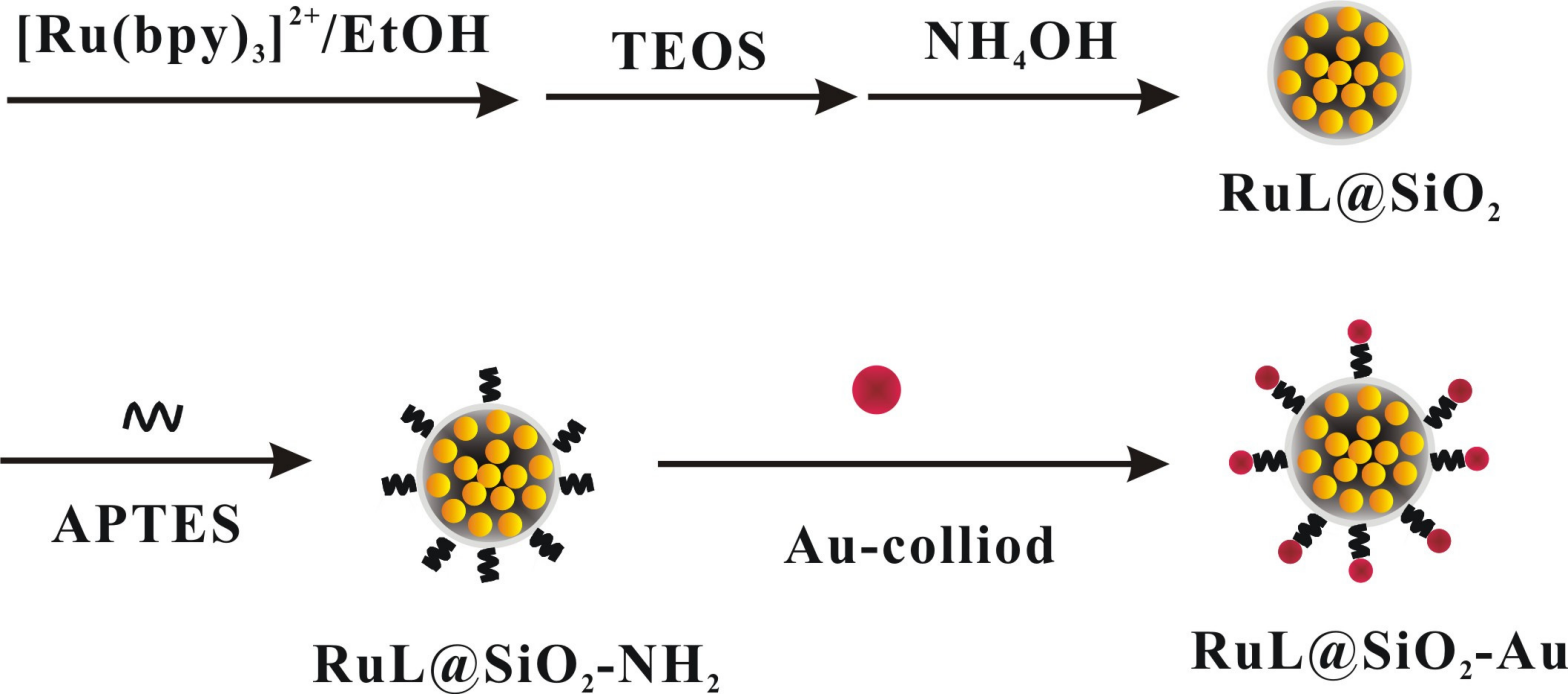
Schematic illustration of the preparing procedures of RuL@SiO_2_ nanoparticles and RuL@SiO_2_-Au composite.

RuL@SiO_2_ nanoparticles were prepared according to the literature [16,28]. In brief, to a mixture of TX-100 (1.77 mL), cyclohexane (7.5 mL), *n*-hexanol (1.8 mL), 0.1 M Ru(bpy)_3_^2+^ (80 μL), tetraethylorthosilicate (TEOS, 100 μL) and water (340 μL) was added NH_4_OH (60 μL, 25%). The mixture was stirred for 24 h, and the generated solid was isolated with acetone, sonicated for 10 min, followed by centrifuging and washing with ethanol and water. Then, the orange RuL@SiO_2_ nanoparticles were collected and dried in vacuum oven. To prepare the RuL@SiO_2_-Au nanoparticles, RuL@SiO_2_ nanoparticles suspension in ethanol (6 mL, 2 mg mL^−1^) was mixed with APTES (200 μL) and vigorously stirred for 30 min. The mixture was centrifuged and washed with water and ethanol to produce aminoterminated RuL@SiO_2_ nanoparticles. A mixture of Au-colloid (2 mL) and APTES-modified RuL@SiO_2_ suspension (1 mL) was then prepared and shaken for 1 h to let Au NPs be absorbed by the surface of the amino-terminated RuL@SiO_2_ nanoparticles. The thus generated RuL@SiO_2_-Au nanoparticles were collected by centrifugation, washed with water and suspended in water. The schematic graph of the fabrication process of RuL@SiO_2_ and RuL@SiO_2_-Au nanoparticles was shown in Scheme 1.

### 2.4. Preparation of RuL@SiO_2_-Au∼RuL-Ab2 and RuL@SiO_2_-Au∼Ab2 (RuL@SiO_2_-Au Composite Nanoparticles Labeled AFP Secondary Antibody)

**Scheme 2.**
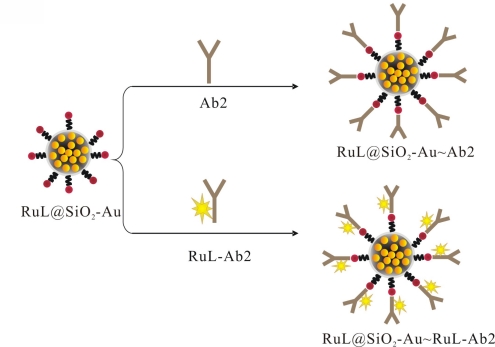
Schematic illustration of the preparing procedures of RuL@SiO_2_-Au∼RuL-Ab2 and RuL@SiO_2_-Au∼Ab2.

The RuL@SiO_2_-Au∼RuL-Ab2 bionanocomposite were prepared according to the literature [17]. In brief, a mixture of RuL@SiO_2_-Au nanoparticles suspension and excess RuL-Ab2 was stirred for 24 h at 4 °C. The mixture was centrifuged and washed with water to obtain the RuL@SiO_2_-Au∼RuL-Ab2 bionanocomposite. The as-prepared bionanocomposite was incubated with BSA to block the unreacted and nonspecific sites, and then centrifuged and washed with PBS, dispersed in 0.1 M PBS (2 mL, pH 7.4) and stored at 4 °C for further use. The RuL@SiO_2_-Au∼Ab2 bionanocomposite was fabricated by using the same procedure, as shown in Scheme 2.

### 2.5. Preparation of the SBAR Sandwich-Type Immunocomplexes

**Scheme 3.**
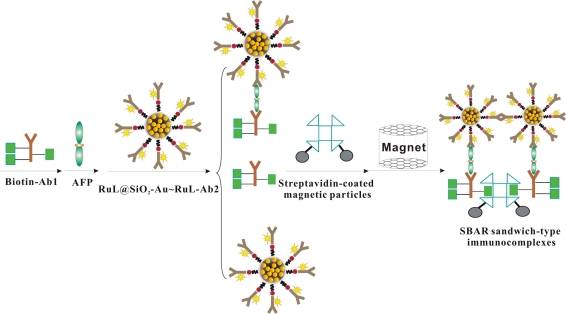
Schematic illustration of the preparing procedures of SBAR sandwich-type immunocomplexes (design and process).

The schematic of the fabrication process is shown in Scheme 3. The immunocomplexes were prepared as follows: a mixture of Biotin-Ab1 (50 μL), different concentrations of AFP (50 μL) and RuL@SiO_2_-Au∼RuL-Ab2 (50 μL) was prepared and held for 10 min at room temperature. After that, streptavidin-coated magnetic particles (SMP, 50 μL) were added into the mixture, allowing the RuL@SiO_2_-Au∼RuL-Ab2-AFP-Ab1-Biotin sandwich type immune-complexes to be captured on the surface of magnetic particles via biotin-streptavidin interaction. Then the sandwich-type immunocomplexes were isolated by a magnet, washed with PBST solution three times, dispersed in PBS (150 μL, pH 7.4) and stored at 4 °C for ECL tests.

### 2.6. ECL Measurements

**Scheme 4.**
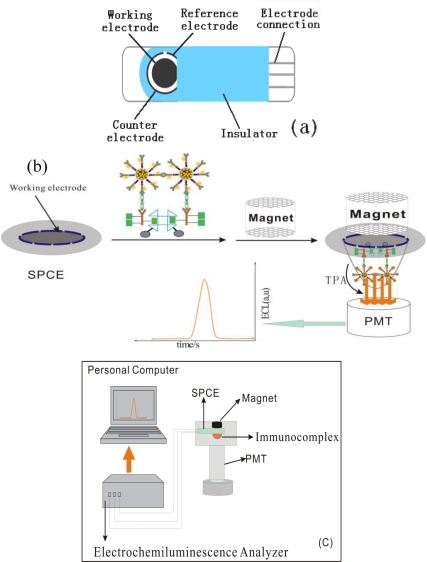
**(a)** Schematic illustration of three-electrode SPCE system; **(b)** The process of ECL measurements; **(c)** the ECL measurement system.

The three-electrode system and ECL measurement process are shown in Scheme 4. For each test, sandwich-type immunocomplex solution (5 μL) prepared with different concentrations of target AFP was attached on the cleaned SPCE surface with a NdFeB permanent magnet, ECL measurements were then performed in PBS (30 μL, pH 7.4) containing 10^−5^ M TPA with a photomultiplier tube voltage of 800 V.

## 3. Results and Discussion

### 3.1. Characterization of RuL@SiO_2_ and RuL@SiO_2_-Au Nanoparticles

**Figure 1.**
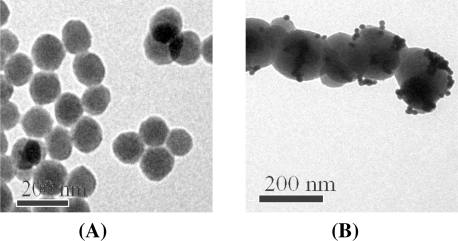
TEM images of **(A)** RuL@SiO_2_; **(B)** RuL@SiO_2_-Au.

In this work, [Ru(bpy)_3_]^2+^-doped silica matrix loaded with Au-NPs, named RuL@ SiO_2_-Au, was prepared as ECL signal amplification labels and immobilization substrates for AFP secondary antibody (Ab2). RuL@SiO_2_ nanoparticles were first fabricated by using the well-established water-in-oil (W/O) microemulsion method. Figure 1(A) shows the TEM image of RuL@SiO_2_ nanoparticles with a uniform size distribution (∼120 nm diameter). Incorporation of RuL molecules inside the silica matrix protects them from the surrounding environment, increases photostability and provides signal enhancement due to an increasing amount of RuL molecules doped per nanoparticle [29]. Furthermore, the ease of assembling functional groups such as amines, thiols and carboxyls on the surface of [Ru(bpy)_3_]^2+^-doped silica nanoparticles enables their use as ideal amplification labels for bioanalysis applications [30]. To immobilize AFP secondary antibody on the RuL@SiO_2_ matrix, the surface of RuL@SiO_2_ nanoparticles was aminoterminated with APTES and further reacted with Au-NPs. Figure 1(B) demonstrates that some individual Au-NPs (∼16 nm diameter) and cluster-shape Au-NPs were successfully assembled on the surface of RuL@SiO_2_ nanoparticles. These attached Au-NPs could provide a biocompatible, accessible matrix for immobilization of AFP secondary antibody.

### 3.2. Optimization of Experimental Conditions

**Figure 2.**
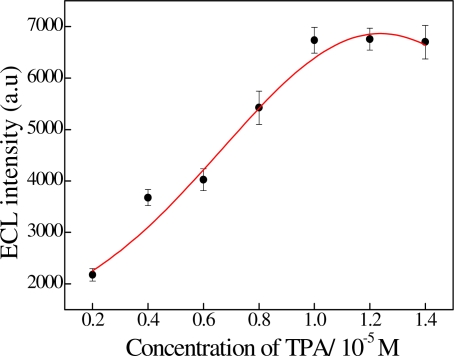
Effect of the different concentrations of TPA on the ECL intensity. Experimental parameters: initial potential = 0.0 V, high potential = 1.2 V, scan rate = 100 mV/s.

The ECL behavior of the sandwiched immunoassay was caused by the TPA and Ru(bpy)_3_^2+^. Thus TPA plays an important role in enhancement of the ECL signal. Furthermore, the enhanced ECL signal was related to the concentration of TPA. As can be seen from Figure 2, the ECL signal increased with increasing concentration of TPA. The effective enhancement occurred at the TPA concentration of 10^−5^ M. Thus 0.1 M of PBS containing 10^−5^ M TPA was selected for our measurements.

### 3.3. ECL Characterization of RuL@SiO_2_-Au∼RuL-Ab2

**Figure 3.**
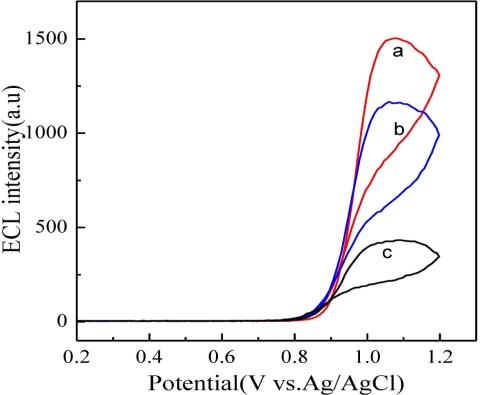
ECL-potential curves of, **(a)** SCMP/Biotin-Ab1/AFP/RuL@SiO_2_-Au∼RuL-Ab2; **(b)** SCMP/Biotin-Ab1/AFP/RuL@SiO_2_-Au∼Ab2; and **(c)** SCMP/Biotin-Ab1/AFP/RuL-Ab2 in pH 7.4 PBS containing 10^−5^ M TPA. Experimental parameters: initial potential = 0.0 V, high potential = 1.2 V, scan rate = 100 mV/s.

Prior to the use of RuL@SiO_2_-Au∼RuL-Ab2 as labels for the preparation of ECL immunosensors, we investigated the ECL performance of RuL@SiO_2_-Au∼RuL-Ab2 in the presence of TPA. For sandwich-type immunosensors, the sensitivity is mainly determined by the sensitivity of the label. In this work, the signal of the ECL immunosensor was mainly from the encapsulated RuL toward TPA oxidation. Since a large number of RuL molecules were incorporated into SiO_2_ nanoparticles, and due to the intrinsic good electrocatalytic activity of RuL toward TPA oxidation, we hypothesized the sensitivity of the ECL immunosensor could be greatly enhanced when RuL@SiO_2_-Au∼RuL-Ab2 was used as recognition element of target AFP and signal amplification label. Figure 3 shows the different modified electrodes in PBS in the presence of 10^−5^ M TPA. It can be seen that only a small ECL intensity change was observed at the RuL-Ab2 modified electrode (curve c). On the contrary, a remarkable ECL intensity increase was observed for the RuL@SiO_2_-Au∼Ab2 modified electrode (curve b) and the largest ECL intensity response occurred at the RuL@SiO_2_-Au∼RuL-Ab2 modified electrode (curve a). These results indicated that a large amount of RuL molecules were incorporated into the silica matrix and RuL-Ab2 could be efficiently captured on the surface of RuL@SiO_2_-Au nanoparticles.

### 3.4. Performance of the ECL Immunosensor

The highly sensitive label of the SBAR sandwich-type immunocomplexes was then used to construct ECL immunosensors for AFP detection. The double-conjugated Ru(bpy)_3_^2+^ sandwich-type immunocomplexes named Biotin-Ab1/AFP/RuL@SiO_2_-Au∼RuL-Ab2 were formed through antigen-antibody interaction in the presence of different concentrations of AFP. They were further interacted with streptavidin-coated magnetic particles (SCMP) and attached on SCPEs by magnet for ECL measurements. Then the ECL response of RuL and TPA was recorded in 0.1 M PBS (pH 7.4) containing 10^−5^ M TPA.

**Figure 4.**
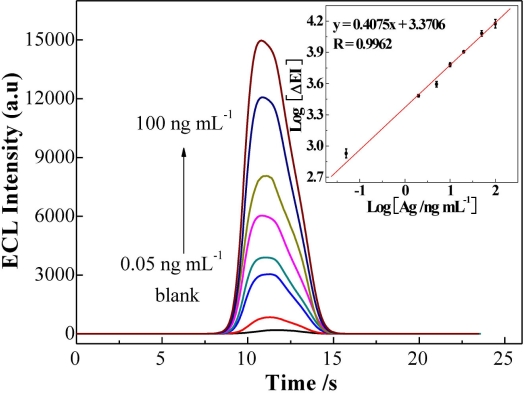
The schematic illustration of the ECL intensity *versus* the concentration of AFP (0.05 to 100 ng mL^−1^) in 0.1 M PBS (pH 7.4) containing 10^−5^ M TPA. Insert: the relationship between Log of ΔECL signal towards log of different AFP concentrations. Experimental parameters: initial potential = 0.0 V, high potential=1.2 V, scan rate = 100 mV/s.

As shown in Figure 4, EI increased with the increasing of AFP concentration ranging from 0.05 to 100 ng mL^−1^. A linear relation between the logarithm of EI and the logarithm of AFP concentration was obtained [Log (ΔEI) = 3.3706 + 0.4075 Log (*c*_AFP_/ng mL^−1^)] with a correlation coefficient R = 0.9962. The detection limit was 0.02 ng mL^−1^ (3σ). Such a low detection limit is better than those of previously reported AFP immunosensors. The comparison of results is shown in Table 1. The improved detection limit may be attributed to two aspects: (1) the high loading level of RuL molecules into the silica nanoparticles improved the detection limit; (2) the large amount of RuL-Ab2 absorbed onto the Au-NPs surface enhanced the access chance of the antibody-antigen interaction, especially when the AFP concentration is very low.

### 3.5. Specificity for the Detection of AFP

**Figure 5.**
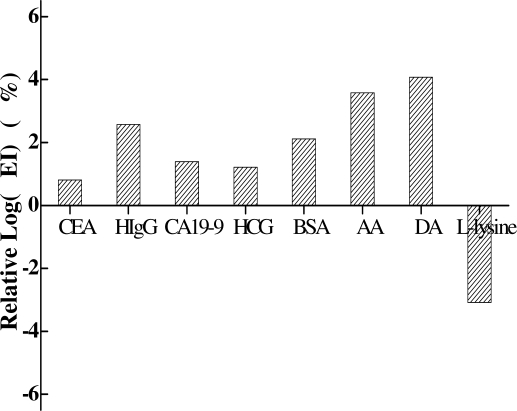
Selectivity analysis of the ECL immunosensor in the presence of different interferents. The concentrations of the interferents were: CEA (10 ng mL^−1^), HIgG (1 μg mL^−1^), CA19-9(10 ng mL^−1^), HCG (10 ng mL^−1^), BSA (1 μg mL^−1^), DA (1 μg mL^−1^), L-lysine (1 μg mL^−1^).

The selectivity of the immunosensor was also tested by adding possible interfering substances in the AFP-mediated sandwich-type immunoreaction. Different immunocomplexes were prepared with AFP (10 ng mL^−1^) or AFP (10 ng mL^−1^) together with the following individual interferent: carcinoembryonic antigen (CEA, 10 ng mL^−1^), human IgG (HIgG, 1 μg mL^−1^), carbohydrate antigen 19-9 (CA19-9, 10 ng mL^−1^), human chorionic gonadotropin antigen (HCG, 10 ng mL^−1^), BSA (1 μg mL^−1^), ascorbic acid (AA, 1 μg mL^−1^), dopamine (DA, 1 μg mL^−1^) and L-lysine (LL, 1 μg mL^−1^). The interference degree was evaluated by comparing the ECL intensity of a mixture of AFP and interfering substance with that of AFP alone. As can be seen from Table 1, less than 5% variation of log(EI) responding to 10 ng mL^−1^ AFP was observed in the presence of different interferents (Figure 5), which demonstrated a good selectivity of the developed ECL immunosensor for AFP detection.

### 3.6. Determination of AFP in Human Serum Samples

In order to investigate the possible application of this immunosensor in clinical analysis, recovery experiments were performed by standard addition methods in human serum. The experimental results were shown in Table 2 and the recovery was in the range from 95% to 110%, which indicated that the developed sensor might be applied for the determination of AFP in human serum for routine clinical diagnosis.

### 3.7. Regeneration

**Figure 6.**
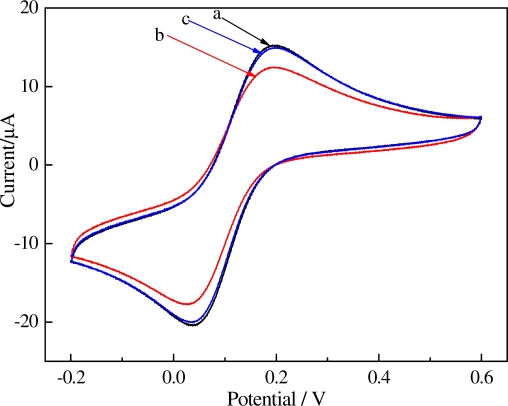
Cyclic voltammograms performed in 1 mM Fe(CN)_6_^3−^ solution (0.1 M KCl) at 100 mV s^−1^ for **(a)** bare; **(b)** SBAR sandwich-type immunocomplexes; **(c)** removing the magnet and rinsing with PBST.

The magnetic probe (SBAR) can be modified and removed from its surface by magnetic field added on the flat bottom of SPCEs. All these steps can renew the electrode surface and make the electrode reusable. As Figure 6 showed cyclic voltammetry (CV) curves of different electrodes (a, bare SPCEs; b, the immunocomplexes were immobilized on the working electrode of SPCEs surface; c, after renewing of SPCEs.) in 1 mM K_3_Fe(CN)_6_ (0.1 M KCl), well-defined CVs, characteristic of diffusion-limited redox processes, are observed at the bare SPCEs [Figure 6(a)]. The peak currents decreased [Figure 6(b)] after SBAR sandwich-type immunocomplexes were immobilized on the working electrode of SPCEs surface because the immunocomplexes would block the electron transfer. After removing the magnet and rinsing the sensor with PBST, the currents was further increased [Figure 6(c)] almost black to bare SPCEs, due to the bulk of immunocomplexes were rinsed out.

## 4. Conclusions

In this paper, a magnetic probe (SBAR) consisting of streptavidin-coated magnetic particles (SCMP) and RuL@SiO_2_-Au∼RuL-Ab2 complexes is used to detect AFP as a model protein. SCMP were used as supporting material for the preparation of the sandwich-type immunocomplexes. A magnetic separation step was then used to isolate the complexes from the unbound components, considerably reducing incubation and washing times. Furthermore, the SBAR sandwich-type immunocomplexes can be modified and removed from its surface by magnetic field added on the flat bottom of SPCEs, therefore, the electrodes, unlike traditional electrochemical immunosensors, do not require complex modification and cleaning steps. In addition, the RuL@SiO_2_-Au∼RuL-Ab2 composite exhibited dual amplification since SiO_2_ could load large amount of reporter molecules (RuL) and gold particles could provide large active surface to load more reporter molecules (RuL-Ab2). Thus the immunosensor can simultaneously realize separation, enrichment and determination, with high sensitivity, which would be valuable for clinical immunoassay for AFP in human serum.

## Figures and Tables

**Table 1.** Comparison of analytical properties of various AFP immunosensors and immunoassays.

**Assay method**	**Linear range (ng mL^−1^)**	**LOD (ng mL^−1^)**	**Detection antibody**	**Reference**
Electrochemistry assay	1.00–500.0	0.8	Au-Alkaline phosphatase-labeled antibody	[31]
	0.50–80.0	0.25	HRP-antibody	[32]
Voltammetric ELISA	0.10–200	0.10	HRP-antibody	[33]
Chemiluminescence	1.0–800	0.23	HRP-antibody	[34]
Photoelectrochemistry	0.05–50	0.04	Label-free	[35]
Electrochemiluninescence	0.05–50	0.03	Ru-silica@Au-antibody	[17]
	0.05–100	0.02	RuL@SiO_2_-Au-RuL-antibody	This work

**Table 2.** The recovery of the proposed immunosensor in human serum.

**Sample number**	**Added (/ng mL^−1^)**	**Found (/ng mL^−1^)**	**Recovery (/%)**
1	0.50	0.51 ± 0.02	102.0
2	1.00	0.97 ± 0.02	97.0
3	5.00	5.28 ± 0.2	105.6
4	10.0	9.8 ± 0.3	98.0
5	50.0	47.6 ± 2.3	95.2

1 Mean value ± SD of three measurements.
